# Early mobilization protocols for critically ill pediatric patients: systematic review

**DOI:** 10.5935/0103-507X.20190038

**Published:** 2019

**Authors:** Taila Cristina Piva, Renata Salatti Ferrari, Camila Wohlgemuth Schaan

**Affiliations:** 1 Programa de Residência Integrada Multiprofissional em Saúde da Criança, Hospital de Clínicas de Porto Alegre, Universidade Federal do Rio Grande do Sul - Porto Alegre (RS), Brasil.; 2 Hospital de Clínicas de Porto Alegre, Universidade Federal do Rio Grande do Sul - Porto Alegre (RS), Brasil.

**Keywords:** Critical care, Child, Early mobilization, Rehabilitation, Intensive care units, pediatric, Cuidados críticos, Criança, Deambulação precoce, Reabilitação, Unidades de terapia intensiva pediátrica

## Abstract

**Objective:**

To describe the existing early mobilization protocols in pediatric intensive care units.

**Methods:**

A systematic literature review was performed using the databases MEDLINE^®^, Embase, SciELO, LILACS and PeDRO, without restrictions of date and language. Observational and randomized and nonrandomized clinical trials that described an early mobilization program in patients aged between 29 days and 18 years admitted to the pediatric intensive care unit were included. The methodological quality of the studies was evaluated using the Newcastle-Ottawa Scale, Methodological Index for Non-Randomized Studies and the Cochrane Collaboration.

**Results:**

A total of 8,663 studies were identified, of which 6 were included in this review. Three studies described the implementation of an early mobilization program, including activities such as progressive passive mobilization, positioning, and discussion of mobilization goals with the team, in addition to contraindications and interruption criteria. Cycle ergometer and virtual reality games were also used as resources for mobilization. Four studies considered the importance of the participation of the multidisciplinary team in the implementation of early mobilization protocols.

**Conclusion:**

In general, early mobilization protocols are based on individualized interventions, depending on the child's development. In addition, the use of a cycle ergometer may be feasible and safe in this population. The implementation of institutional and multidisciplinary protocols may contribute to the use of early mobilization in pediatric intensive care units; however, studies demonstrating the efficacy of such intervention are needed.

## Abstract (pt)

**Objetivo:**

Descrever os protocolos existentes de mobilização precoce nas unidades de terapia intensiva pediátrica.

**Métodos:**

Trata-se de uma revisão sistemática da literatura cuja busca foi realizada nas bases MEDLINE^®^, Embase, SciELO, LILACS e PeDRO, sem restrição para data e idioma. Foram incluídos estudos observacionais e ensaios clínicos randomizados e não randomizados, que descrevessem um programa de mobilização precoce em pacientes admitidos na unidade de terapia intensiva pediátrica, com idades entre 29 dias a 18 anos. A qualidade metodológica dos estudos foi avaliada por meio das ferramentas *Newcastle-Ottawa, Methodological Index for Non-Randomized Studies* e da colaboração *Cochrane*.

**Resultados:**

Foram identificados 8.663 estudos, sendo 6 incluídos nesta revisão. Três estudos descreveram a implementação de programa de mobilização precoce, incluindo atividades como mobilização passiva progressiva, posicionamento, discussão das metas de mobilização com a equipe, além de contraindicações e critérios de interrupção. Cicloergômetro e jogos de realidade virtual também foram usados como recursos para mobilização. Quatro estudos consideram a importância da participação da equipe multiprofissional na implementação dos protocolos de mobilização precoce.

**Conclusão:**

De modo geral, os protocolos de mobilização precoce são baseados em intervenções individualizadas, conforme o desenvolvimento da criança. Além disso, o uso do cicloergômetro pode ser viável e seguro nesta população. A implementação de protocolos institucionais e multiprofissional pode contribuir para a prática da mobilização precoce nas unidades de terapia intensiva pediátrica, no entanto são necessários estudos que comprovem a eficácia da intervenção.

## INTRODUCTION

The initial goal in the management of critically ill patients in intensive care units (ICUs) is to maintain maximal hemodynamic and ventilatory stability.^(1)^ In recent years, mortality in pediatric ICUs has significantly decreased, but the proportion of children who developed some degree of limitation after discharge has increased.^(2,3)^ The consequent immobilization, together with other risk factors such as sepsis, hyperglycemia, prolonged length of hospital stay, and use of corticosteroids, benzodiazepines and neuromuscular blocking agents, may be related to functional limitation,^(4)^ decreased muscle mass and strength, alterations in skin integrity, withdrawal and *delirium*.^(5,6)^

In this context, interventions such as early mobilization, initiated immediately after ICU patient stabilization, should be considered in the patient rehabilitation process.^(7-11)^ In adults, early mobilization is associated with short- and long-term positive outcomes, such as improvement in peripheral muscle strength,^(12)^ mobility and days out of the hospital.^(13)^ In children, studies are recent,^(14-16)^ but the evidence shows that early mobilization is feasible and safe.^(17,18)^

The lack of protocols and of knowledge of the multidisciplinary team, the concern with patient safety, the level of sedation and the availability of professionals and resources are important barriers to the use of early mobilization in pediatric ICUs.^(19,20)^ Thus, the objective of this review was to describe the early mobilization protocols available for the pediatric population, analyzing the proposed activities, the necessary resources and the professionals involved. The systematization of these protocols may contribute to a better understanding and recommendation of this practice, aiming to reduce associated morbidity and to achieve functional recovery of children and adolescents through the implementation of safe practices in pediatric ICUs.

## METHODS

This was a systematic literature review that followed the recommendations of the PRISMA Statement^(21)^ and is registered in the International prospective register of systematic reviews (PROSPERO) under number CRD42017068238.

### Eligibility criteria

Observational studies and randomized, nonrandomized or quasi-experimental clinical trials describing early mobilization protocols in the pediatric ICU for children and adolescents aged between 29 days and 18 years were included. Early mobilization was defined as any mobility exercise, whether passive or active, initiated as early as possible during the stay in the pediatric ICU and included passive, active-assisted or active exercises; bed mobility activities (example: changing positions and sitting); transfers; orthostasis; stationary gait and/or ambulation; and mobilization with a cycle ergometer or virtual reality games (Nintendo Wii(tm) or Xbox 360 Kinect(tm)). The time of beginning of mobilization, based on admission, was not considered an inclusion criterion. Studies published in English, Portuguese or Spanish were included.

### Search strategy and selection of studies

The search was performed in the databases MEDLINE^®^ via PubMed^®^, Embase, Physiotherapy Evidence Database (PEDro), Latin American & Caribbean Health Sciences Literature (LILACS) and Scientific Electronic Library Online (SciELO). A manual search was also performed in the references of published studies on the subject.

The search strategy comprised keywords and synonyms for the intervention "early mobilization" and for the study population "children and adolescents in intensive care". The search was performed using MeSH terms and synonyms, without restrictions for date or language, until March 2017, and updated in January 2018. The complete PubMed^®^ search strategy is provided in appendix A.

The titles and abstracts of the articles identified in the search were analyzed by 2 independent reviewers, according to the inclusion and exclusion criteria. In the next phase, the same reviewers performed a full reading of the articles selected to independently assess if they met the eligibility criteria. Articles with insufficient information in the abstract were also selected for full reading. In cases of disagreement, a third evaluator was consulted.

### Extraction and analysis of data

The data were extracted independently by the reviewers using a standardized table, which comprised the sample characterization, description of the early mobilization protocol (beginning of mobilization, activity performed, resources used, duration, frequency and progression), the professionals involved and the main results found. Data were analyzed descriptively.

### Assessment of the risk of bias

The methodological quality was evaluated in a descriptive and independent manner by the same 2 reviewers. The methodological quality of the observational studies was evaluated by the Newcastle-Ottawa Scale (NOS); prospective studies were evaluated using the tool for cohort studies, and retrospective studies were evaluated using the tool for case-control studies, considering 3 aspects: group selection (zero - 4 points), quality of the adjustment for the confounders (zero - 2 points) and evaluation of the exposure or outcome of interest in the study (zero - 3 points), totaling 9 points, which represents high methodological quality.^(22)^ Randomized controlled clinical trials were evaluated as recommended by the Cochrane Collaboration through the following items: random sequence generation, allocation concealment, blinding of outcome assessment, intent-to-treat analysis and description of losses and exclusions.^(23)^ Nonrandomized studies were evaluated according to the Methodological Index for Non-Randomized Studies (MINORS), which comprises 12 items, with the first 8 being applicable to noncomparative studies and scored as 0 (unreported), 1 (reported but inadequate) or 2 (reported and adequate), totaling 16 points.^(24)^

## RESULTS

**Figure 1:**
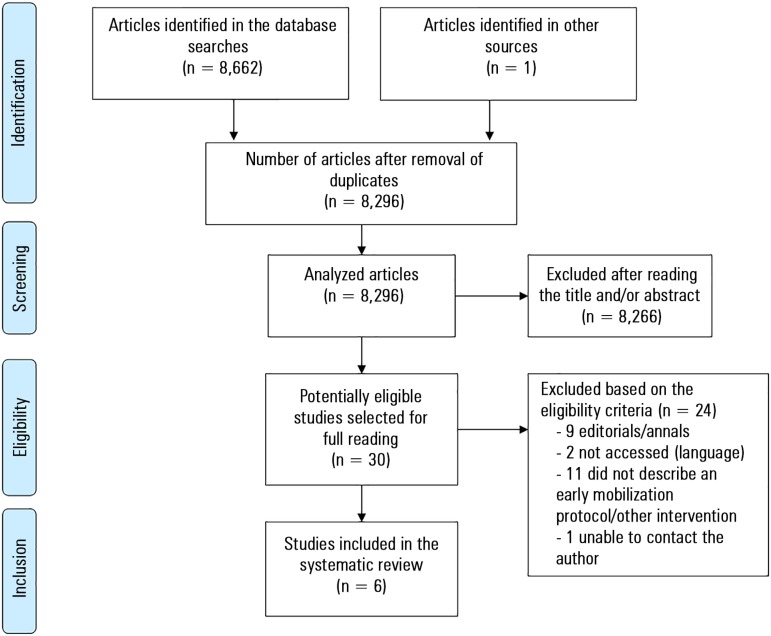
Flowchart of the studies included in the systematic review.

Six of the 8,663 studies identified were included in this systematic review (Figure 1). The final sample included 2 prospective observational studies,^(17,25)^ 1 retrospective observational study,^(26)^ 2 quasi-experimental studies^(14,15)^ and 1 randomized controlled trial,^(18)^ totaling 394 patients, with a mean age of 8 years, ranging from children under 1 year to 16 years of age. The reason for admission to the pediatric ICU varied among the studies, including clinical and surgical causes. The characteristics of the included studies are provided in table 1.

**Table 1:** Characteristics of the included studies

Author	Drawing of a sample	Age (years)	Sample characteristics	Objective
Abdulsatar et al.^(14)^	Quasi-experimental N = 12 (Intervention N = 8)	11 (3 - 16)*	Children and adolescents (3 - 18 years) in the pediatric ICU with expected length of stay > 48 hours. PRISM III 9.5 (0 - 21)* PCPC 1 (1, 2)* and POPC 1 (1,3)*	Assess the viability and safety of exercise with virtual reality games in critically ill children
Choong et al.^(15)^	Quasi-experimental N = 31 (Intervention N = 25)	11 (6 -14)*	Patients (3 - 17 years) with expected length of stay in the pediatric ICU > 24 hours. PRISM III 6 (0 - 8)† PCPC 3 (1 - 4)† and POPC 2 (1 - 5)†	Evaluate the viability and safety of the implementation of 2 rehabilitation methods based on passive and active in-bed mobilization in critically ill children
Wieczorek et al.^(17)^	Prospective N = 100 mobilization N = 100 pre-implementation	7.7 ± 5.4‡	Children and adolescents (< 17 years) admitted to the pediatric ICU for ≥ 3 days PRISM 5,4 (4,5)‡	Determine the safety and feasibility of an early mobilization program in the pediatric ICU
Choong et al.^(18)^	Pilot RCT N = 20 intervention N = 10 control	8 (5 - 14)† intervention 9 (6 - 11)† control	Children and adolescents (3 - 17 years) with expected length of stay in the pediatric ICU > 48 hours Intervention: PRISM III = 8 (6 - 13) PCPC = 1 (1-3) and POPC = 1 (1 - 2) Control: PRISM III = 10 (7 - 16) PCPC = 2 (1 - 3) and POPC = 2 (1 - 3)	Determine the feasibility of a study on the efficacy of early mobilization with a cycle ergometer combined with physical therapy in the functional recovery of critically ill pediatric patients
Tsuboi et al.^(25)^	Prospective N = 34 mobilization N = 23 pre-implementation	1.1 (0.58 - 6.16)†	Pediatric patients (< 16 years) after liver transplantation. PIM2(%) 3.2 (1.2 - 3.7)* PELD 6 (0 - 12)*	Assess the impact of an early mobilization program in the pediatric ICU after liver transplantation
Betters et al.^(26)^	Retrospective N = -74	4.4 (1.8 - 12.8)†	Patients under MV, cooperative and alert. Sedation level > 2 according to scale used	Describe the creation and implementation of an early mobilization protocol for pediatric patients under MV

ICU - intensive care unit; PRISM - Pediatric Risk of Mortality; PCPC - Pediatric Cerebral Performance Category; POPC - Pediatric Overall Performance; RCT - randomized controlled trial; PIM2 - Pediatric Index of Mortality; PELD - Pediatric End-Stage Liver Disease; MV - mechanical ventilation.

* Median (minimum-maximum);

† median (interquartile range 25-75);

‡ mean ± standard deviation.

The early mobilization protocols used are provided in table 2. Three studies described the implementation of an early mobilization program.^(17,25,26)^ The first study described an interdisciplinary mobilization program called *PICU Up!*, consisting of 3 progressive levels based on the patient's condition, ventilatory parameters and sedation level defined daily during rounds. The protocol included routines such as lighting, positioning, change in position, physical therapy and occupational therapy, sitting, leaving the bed and walking, and daily assessment of *delirium*. Activities were implemented by the nursing team and other professionals involved and planned according to the child's needs.^(17)^

**Table 2:** Characteristics of early mobilization protocols in pediatric patients

Author	Beginning	Contraindications	Early mobilization protocol	Main results
Abdulsatar et al.^(14)^	9.5 (1 - 56)* days	Hemodynamic instability; deep sedation; contraindication for mobilization (e.g., surgery in ULs); severe cognitive or functional disability (POPC and PCPC ≥ 4); on life support	Interactive videogame Nintendo Wii ™ Boxing - Sport Pack 2 times/day, minimum 10 minutes	Increased movement of the ULs versus the remainder of the day (p = 0.049) No difference in grip strength (p = 0.20) 75% did not complete the 2-day intervention protocol due to excessive sedation, pediatric ICU transfer or refusal by the parents/patient Limitation of intervention viability due to restricted number of eligible patients
Choong et al.^(15)^	4 (2 -10)* days	Hemodynamic and ventilatory instability; active patients or at their baseline level of functionality; imminent risk of death; on life support; cerebral edema, elevated intracranial pressure, unstable spinal cord injuries; musculoskeletal injuries, surgical contraindications and deformities Interruption criteria: bradycardia, tachycardia, hypotension, persistent hypertension, SpO_2_<85% or increased work of breathing; pain or discomfort; drain and tube dislodgement	Interactive videogame for cooperative and conscious patients. Nintendo Wii™ Sport Pack and Mario Kart Cycle cyclometer passive exercise for LLs for noncooperative patients Ex N’Flex EF-300 (3 - 7 years) MOTOmedLetto2 (8 - 17 years) Day 1: 10 - 20 minutes Day 2: 20 minutes	Passive mobilization with cycle ergometer increased the activity of the LLs (p <0.001) Safe when applied to noncooperative children Activities with interactive videogames are viable only in a minority of children and did not increase the movement of the ULs (p> 0.05)
Wieczorek et al.^(17)^	First 72 hours after admission	ECMO; unstable fracture; thorax or abdomen exposed; medical orientation Break/reevaluation criteria: 20% change in HR, BP or RR; 15% decrease in SpO_2_; Need to increase FiO_2_ by 20%; increase in ETCO_2_ by 20%; work of breathing; new arrhythmia; change in mental state; agitation; concern with OTT/TQT, vascular access or EVD	LEVEL 1 (MV FiO_2_> 0.6 or PEEP> 8, difficult intubation, recent TQT, acute neurological event, vasopressor, sedation or SBS -3 and -2): Lights on 9am - 11pm Television 2 hours/day > 2 years Elevated headboard ≥ 30 Change in position Positioning Physical therapy initiation Evaluation by the occupational therapist after 72 hours LEVEL 2 (MV FiO_2_ ≤ 0.6 or PEEP ≤ 8 and SBS -1 and +3 or NIV FiO_2_ > 0.6, dialysis/renal replacement therapy or femoral access): Positive touch Sitting up in bed 3 times/day Consider out of bed to chair and/or ambulation Assessment by the speech pathologist Evaluation of *delirium* 2 times/day. LEVEL 3 (NIV FiO_2_ ≤ 0.6 or baseline pulmonary support or external ventricular drain and SBS -1 and +3): Out of bed to chair 3 times/day or sitting up in bed Ambulation 2 times/day if trunk control present	Increase in the number of physical therapy and occupational therapy consultations with the implementation of the early mobilization program The mean number of mobilization activities per patient on the 3rd day doubled from 3 (2 - 5)† to 6 (3 - 7.5)^b^ (p < 0.01)
Choong et al.^(18)^	2 (1 - 4)† days	Hemodynamic, ventilatory and/or neurological instability; surgical contraindications Interruption criteria: SpO_2_ < 88% despite an increase in FiO_2_; tachycardia, bradycardia and persistent hypotension, arrhythmia; increase in blood pressure 25%; increased work of breathing; discomfort or pain	Intervention: standard treatment + cycle ergometer RT300 Supine Cycle Ergometer 30 minutes - 5 times/week Control: standard treatment according to the institutional routine of early mobilization‡ Participants were mobilized at increasing levels individually according to the necessary assistance and could involve activities such as positioning, passive exercises, active exercises, muscle strengthening, transfers, changes in position, sitting periods	Early mobilization is safe and viable In-bed mobilization with a cycle ergometer can optimize the duration and intensity of mobilization in previously healthy children with pre-existing functional limitations
Tsuboi et al.^(25)^	From the 1st PO day	Hemodynamic instability; PO immediately after thoracic or abdominal surgery; intracranial hypertension; cervical spinal instability	Daily planning of the level of mobilization for each patient with the team: range-of-motion exercises; sitting on the bed; transfer to a chair; orthostasis; ambulation§	Increase in the proportion of patients who received physical therapy after the implementation of the early mobilization program (p < 0.001) No difference in the time of intubation, length of stay on the pediatric ICU and length of hospital stay The mobilization was well tolerated and safe
Betters et al.^(26)^	Daily assessment of patients under MV	Absolute: high-frequency oscillatory ventilation; neuromuscular blocking agent; difficult airway; unstable TBI Relative: FiO_2_ > 0.5 or rapid increase; PEEP > 8; sedation level < 2; hemodynamic instability; vertebral injury	Active mobilization of patients under MV 10 - 60 minutes/day according to tolerance	Significant difference in the professionals’ perception about mobilization Increased number of consultations The implementation of a multidisciplinary protocol and the training of the team enabled the early mobilization of pediatric patients under MV in the pediatric ICU

ULs - upper limbs; POPC - Pediatric Overall Performance Category; PCPC - Pediatric Cerebral Performance Category; ICU - intensive care unit; SpO_2_ - peripheral oxygen saturation; LLs - lower limbs; ECMO - extracorporeal membrane oxygenation; HR - heart rate; BP - blood pressure; RR - respiratory rate; FiO_2_ - inspired fraction of oxygen; ETCO_2_: end-tidal carbon dioxide; OTT - orotracheal tube; TQT - tracheostomy; EVD - external ventricular drain; MV - mechanical ventilation; PEEP - positive end-expiratory pressure; SBS - State Behavioral Scale; NIV - noninvasive ventilation; PO - postoperative; TBI - traumatic brain injury.

* Median (minimum-maximum);

† median (interquartile range);

‡ http://links.lww.com/pcc/a529;

§ patients under MV: range-of-motion exercises.

The second study analyzed an early mobilization program implemented in a sample of children and adolescents in a pediatric ICU after liver transplantation. One of the elements of this program corresponded to daily planning of mobilization goals by the multidisciplinary team for each patient, involving range-of-motion exercises, sitting, transfer to a chair, orthostasis and ambulation. In patients undergoing invasive mechanical ventilation, only range-of-motion exercises were considered.^(25)^ The third study defined as early mobilization the active mobilization of patients on mechanical ventilation, according to the proposed mobility and development goals for their age.^(26)^

The viability and safety of interactive videogames (Nintendo Wii(tm)) for patients in the pediatric ICU were evaluated in 2 studies.^(14,15)^ A pilot 2-day intervention protocol was performed twice a day for 10 minutes or more.^(14)^ The chosen game was Wii(tm) Boxing*,* which stimulated the active movement of the upper limbs, required minimal manual dexterity and could be performed while lying on the bed; however, it depended on the child's cooperation and level of consciousness. In that study, 75% of the patients included did not complete the 2-day intervention protocol due to excessive sedation, pediatric ICU transfer or refusal by the parents/child. Of the 8 patients included, 4 were under mechanical invasive ventilation during the intervention. Subsequently, the intervention with Nintendo Wii(tm) was compared with intervention with a cycle ergometer, according to the level of consciousness and cognitive ability of the child.^(15)^ In active and conscious patients, active mobilization was stimulated through interactive games with Nintendo Wii(tm) (Sport Pack and Mario Kart), and in uncooperative patients, due to changes in the level of consciousness due to sedation and/or cognitive age, a cycle ergometer passive exercise for the lower limbs was used. The protocol consisted of 2 days of intervention, lasting 10 to 20 minutes on the first day and 20 minutes on the second day.

The use of a cycle ergometer was also evaluated in conjunction with physical therapy in a recent clinical trial by Choong et al.^(18)^ The intervention lasted 30 minutes and was performed 5 times a week. The median age of the randomized patients was 8 years in the intervention group and 9 years in the control group. This study confirmed that early mobilization is safe and feasible in pediatric patients and that mobilization with a cycle ergometer can optimize the duration and intensity of the intervention.

Regarding the professionals involved, 4 studies reported the involvement of a multidisciplinary team in the promotion of early mobilization, involving, in addition to the physical therapist, the nursing team, physicians, occupational therapists and speech therapists.^(17,18,25,26)^

The beginning of early mobilization varied between the studies, from the first to the 56th day of the hospital stay. In a study by Wieczorek et al.,^(17)^ mobilization began in the first 72 hours of admission to the pediatric ICU, similar to a study by Choong et al.,^(18)^ with a median of 2 (1 - 4) days. In post-liver transplant patients, 65% of the sample was mobilized in the first 72 hours after admission.^(25)^ In a study by Betters et al.,^(26)^ mobilization occurred regardless of the length of hospital stay. The patients were evaluated in the first 72 hours of admission and reassessed daily, according to the eligibility criteria, as the intervention depended on the child's cooperation.

The safety of early mobilization was assessed based on the occurrence of adverse events. The intervention was safe in the 6 studies included, and no incident related to mobilization was recorded.

The methodological quality of the observational studies ranged from 2 to 7 points. The main limitations were the limited sample size, the presence of the outcome of interest at the beginning of the study and patient follow-up. The only included randomized clinical trial showed a low risk of bias, as did the quasi-experimental studies, with a total of 12 points. The main limitation of the studies was the blinding of the evaluators given that the main outcome of interest was the viability of the intervention (Table 3).

**Table 3:** Assessment of the risk of bias of the included studies

**Observational studies (NOS)†**									
**Author**		**Selection**		**Comparability**		**Outcome/Exposure:**		**NOS score**	
Wieczorek et al.^(17)^		***		**		**		7	
Tsuboi et al.^(25)^		***		**		**		7	
Betters et al.^(26)^		*		NA		*		2	
**Nonrandomized clinical trials (MINORS)‡**									
**Author**	**Clear objective**	**Inclusion of consecutive patients**	**Prospective data collection**	**Appropriate outcomes**	**Impartial outcome assessment**	**Appropriate follow-up**	**Loss of less than 5%**	**Calculation of the study size**	**MINORS score**
Abdulsatar et al.^(14)^	2	2	2	2	0	2	1	1	12
Choong et al.^(15)^	2	2	2	2	0	2	1	1	12
**Randomized controlled trial (Cochrane Risk of Bias Tool)**									
**Author**	**Random sequence generation **		**Allocation concealment**		** Blinding**		** Description of losses and exclusions**		**Intention-to-treat analysis**
Choong et al.^(18)^	Yes		Uncertain bias		Not applicable		Yes		Yes

† the categories group selection and evaluation of outcome/exposure can receive a maximum of 1 star (*) for each item evaluated corresponding to 4 and 3 points, respectively. The category comparability between groups can receive a maximum of 2 stars for the evaluated item. When the criterion was considered not applicable to the study, no score was assigned;

‡ zero: unreported; 1: reported and inadequate; or 2: reported and adequate, totaling 16 points. NOS - Newcastle-Ottawa Scale; MINORS - Methodological Index for Non-Randomized Studies.

## DISCUSSION

The objective of this systematic review was to describe and analyze the early mobilization protocols in pediatric intensive care; despite limited evidence, the intervention is viable and safe in this setting. In general, in the protocols analyzed, the activities are planned individually and based on the child's development. Resources such as a cycle ergometer and virtual reality games can also be considered in this population.

Studies on early mobilization in the pediatric population are recent. The studies included in this review were published in the last 5 years. Early mobilization has been implemented in some pediatric ICUs, especially in countries such as Canada and the United States. In 2011, in 6 Canadian pediatric ICUs surveyed, less than 10% of patients were mobilized early (< 48 hours), and only 2 ICUs had mobilization guidelines.^(27)^ In a recent study, 77% of patients admitted to a Canadian pediatric ICU were mobilized within 72 hours of admission,^(18)^ similar to a study by Wieczorek et al.^(17)^ (76%). The implementation of institutional protocols, as observed in these recent studies, may facilitate the evaluation and identification of suitable patients and enable mobilization initiation as early as possible.

The interdisciplinary program for early mobilization described by Wieczorek et al.^(17)^ has 3 progressive levels of mobilization, according to clinical and ventilatory variables, and establishes objective criteria in case of a need to break or interrupt the intervention. Programs such as these are able to guide the use of mobilization in the ICU.^(28)^ Practice recommendations for early mobilization in critically ill pediatric patients, prepared by a multidisciplinary group with experience in the field, were recently published.^(29)^

Considering the use of resources to facilitate early mobilization in critically ill pediatric patients, passive mobilization with a cycle ergometer was feasible and safe in most patients, increasing movement of the lower limbs.^(14)^ A recently published study by Choong et al.^(18)^ assessed the efficacy of a cycle ergometer combined with physical therapy in the mobilization of children and adolescents - this is the first randomized controlled trial in this population. It was possible to observe that mobilization with a cycle ergometer can be implemented starting in the first days of admission to the pediatric ICU (1.5 (1 - 3) days in the treatment group *versus* 2.5 (2 - 7) days in the control group). Notably, all patients were mobilized according to the institutional guidelines for mobilization.^(29)^

Regarding interactive videogames (Nintendo Wii(tm)), their use was feasible in only a minority of children in the pediatric ICU, with conflicting results regarding the activity level. Movement of the upper limbs was greater during intervention with the Wii(tm) than throughout the rest of the day.^(14)^ However, in the second study analyzed, there was no increase in the movement of the upper limbs compared to the 20 minutes of highest activity of the day.^(15)^ This finding can be justified because the levels of activity when using videogames are highly variable, depending on the game used and the child's level of understanding and motivation.^(30)^

The term "early mobilization" refers to the rehabilitation of critically ill patients initiated immediately after hemodynamic and respiratory stabilization; the patients may also be undergoing invasive mechanical ventilation and/or using vasopressors.^(31)^ The time of early mobilization initiation varied between the studies analyzed. Currently, there is no consensus on when to begin the intervention. However, the complications related to the immobility of critically ill patients are clearly described in the literature. The loss of muscle mass in adults is still observed as early as the first week of ICU admission.^(32)^ A reduction of 9.5% in quadriceps femoris muscle thickness was observed on the fifth day of admission in children under mechanical ventilation.^(33)^ This reinforces the need for intervention to be started as soon as possible to prevent well-known complications.

Early mobilization may also reduce the occurrence of *delirium* in critically ill patients. The standardization of sedation in pediatric patients undergoing mechanical ventilation and the implementation of an early mobilization program reduced the monthly average prevalence by 8%.^(34)^ The mobilization protocol consisted of 5 progressive levels, similar to the protocol proposed by Wieczorek et al.^(17)^

Of the 6 studies included, 4 reported the involvement of a multidisciplinary team in the early mobilization implementation process. The studies emphasize that the daily and individualized discussion of the intervention goals with other members of the multidisciplinary team is essential for the promotion of mobilization. The optimization of sedation should also be discussed within the team, considering the safety and comfort of the child.^(19,35)^ Given that the main barriers observed in the studies were excessive sedation, number of professionals, associated workload (physical therapists and occupational therapists) and availability of appropriate materials, rounds and checklists can facilitate interprofessional communication and help in the promotion of early mobilization. In addition, the formation of working groups and training and education activities for the care team are important for promoting the use of early mobilization in pediatric ICUs.^(16,17)^

The involvement and participation of the family, item "F" of the ABCDEF bundle,^(36)^ also seem to be facilitating tools in the promotion of early mobilization in pediatric patients, offering comfort to and improving communication with the child and active participation of the family in the care.^(20,26)^

The studies included in this systematic review are methodologically heterogeneous and exhibited wide variability in terms of study populations. The primary reasons for admission to the pediatric ICU involved several clinical conditions and a wide age range. The age of patients in the pediatric ICU can vary from 29 days to 14 or 18 years, according to hospital routines. It is expected that older children are more capable of early mobilization due to cognitive and functional maturity and tolerance to lower levels of sedation.^(37)^ In addition, the prevalence of children admitted to pediatric ICUs with complex chronic conditions should be considered (83.9%);^(38)^ in 1 of the included studies, 70% of patients had a preexisting chronic condition,^(18)^ which may hinder early mobilization.

The available publications on early mobilization in the pediatric population are limited to studies with Level 2 evidence (Oxford Center of Evidence-Based Medicine), while in adults, there is Level 1 evidence on the efficacy of mobilization of critically ill patients in functional recovery.^(29)^ Observational, quasi-experimental studies were included in this review, and only 1 randomized controlled trial was identified. Two ongoing clinical trials were located at *ClinicalTrials.gov* (NCT02958124) (NCT02209935).

### Limitations

Although the present review has strictly followed the PRISMA recommendations and conducted a wide search to identify all published studies, there were some limitations that should be noted. First, due to the lack of intervention studies, observational and nonrandomized or quasi-experimental clinical trials were also included. Another important point was that interventions could vary according to the child's development and level of cooperation, which may influence outcomes and hinder comparisons. Finally, in addition to the methodological differences, the small number of published studies and the sample size stand out, which suggests the need for further studies with a larger number of patients, adequate follow-up time and greater methodological rigor.

## CONCLUSION

The early mobilization protocols are based on individualized interventions, planned according to the child's development. The use of a cycle ergometer as a resource for mobilization may increase the movement of children and adolescents in the pediatric intensive care unit, while the feasibility of using interactive videogames is limited in this population due to their level of cooperation. Despite the evidence available to date and the low methodological rigor of the included articles, the implementation of multidisciplinary protocols seems to be a viable tool for the promotion of early mobilization in pediatric intensive care. However, further studies are needed with standardized intervention protocols and randomized clinical trials to evaluate the efficacy of early mobilization in this population.

## Appendix

**Appendix A:** Search strategy used in PubMed^®^

#1	*("Intensive Care Units, Pediatric" OR "Intensive Care Units, Pediatric" OR “Pediatric Intensive Care Units” OR "Intensive Care Units" OR "Intensive Care Units" OR “Care Unit, Intensive” OR “Care Units, Intensive” OR “Intensive Care Unit” OR “Unit, Intensive Care” OR “Units, Intensive Care” OR "Critical Care" OR “Critical Care” OR “Care, Critical” OR “Intensive Care” OR “Care, Intensive” OR “Surgical Intensive Care” OR “Care, Surgical Intensive” OR “Intensive Care, Surgical” OR "Critical Illness" OR "Critical Illness" OR “Critical Illnesses” OR “Illness, Critical” OR “Illnesses, Critical” OR “Critically Ill”) *
#2	*(Pediatrics OR Infant OR Infants OR “Child, Preschool” OR “Preschool Child” OR “Children, Preschool” OR “Preschool Children” OR Child OR Children OR Adolescent OR Adolescents OR Adolescence OR Teens OR Teen OR Teenagers OR Teenager OR Youth OR Youths OR “Adolescents, Female” OR “Adolescent, Female” OR “Female Adolescent” OR “Female Adolescents” OR “Adolescents, Male” OR “Adolescent, Male” OR “Male Adolescent” OR “Male Adolescents”)*
#3	*(“Early Ambulation” OR “Ambulation, Early” OR “Accelerated Ambulation” OR “Ambulation, Accelerated” OR “Early Mobilization” OR “Mobilization, Early” OR “Exercise Therapy” OR “Therapy, Exercise” OR “Exercise Therapies” OR “Therapies, Exercise” OR Rehabilitation OR Habilitation OR “Physical Therapy Modalities” OR “Modalities, Physical Therapy” OR “Modality, Physical Therapy” OR “Physical Therapy Modality” OR “Physiotherapy (Techniques)” OR “Physiotherapies (Techniques)” OR “Physical Therapy Techniques” OR “Physical Therapy Technique” OR “Techniques, Physical Therapy” OR “Neurological Physiotherapy” OR “Physiotherapy, Neurological” OR “Neurophysiotherapy” OR "virtual rehabilitation" OR "video game" OR "passive cycling exercise" OR "passive cycle ergometer")*
#4	#1 *AND* #2 *AND* #3
